# Community Functional Responses to Soil and Climate at Multiple Spatial Scales: When Does Intraspecific Variation Matter?

**DOI:** 10.1371/journal.pone.0111189

**Published:** 2014-10-20

**Authors:** Andrew Siefert, Jason D. Fridley, Mark E. Ritchie

**Affiliations:** Department of Biology, Syracuse University, Syracuse, New York, United States of America; Fred Hutchinson Cancer Research Center, United States of America

## Abstract

Despite increasing evidence of the importance of intraspecific trait variation in plant communities, its role in community trait responses to environmental variation, particularly along broad-scale climatic gradients, is poorly understood. We analyzed functional trait variation among early-successional herbaceous plant communities (old fields) across a 1200-km latitudinal extent in eastern North America, focusing on four traits: vegetative height, leaf area, specific leaf area (SLA), and leaf dry matter content (LDMC). We determined the contributions of species turnover and intraspecific variation to between-site functional dissimilarity at multiple spatial scales and community trait responses to edaphic and climatic factors. Among-site variation in community mean trait values and community trait responses to the environment were generated by a combination of species turnover and intraspecific variation, with species turnover making a greater contribution for all traits. The relative importance of intraspecific variation decreased with increasing geographic and environmental distance between sites for SLA and leaf area. Intraspecific variation was most important for responses of vegetative height and responses to edaphic compared to climatic factors. Individual species displayed strong trait responses to environmental factors in many cases, but these responses were highly variable among species and did not usually scale up to the community level. These findings provide new insights into the role of intraspecific trait variation in plant communities and the factors controlling its relative importance. The contribution of intraspecific variation to community trait responses was greatest at fine spatial scales and along edaphic gradients, while species turnover dominated at broad spatial scales and along climatic gradients.

## Introduction

Understanding and predicting how communities respond to environmental variation is a central goal of ecology, and ecologists are increasingly adopting trait-based approaches to study these responses [1]–[3]. Because an organism’s functional traits directly influence its responses to and effects on the environment [4], information about the traits of individuals in a community (i.e. community trait distributions) offers insights into community assembly mechanisms and can be used to predict community composition and ecosystem functioning [3], [5], [6]. Understanding how community trait distributions, particularly community-weighted mean trait values (CWMs), respond to environmental variation has therefore become a major focus in community ecology [5], [7], [8].

Community trait distributions may change along environmental gradients through a combination of species turnover (changes in species presence and relative abundance) and intraspecific trait responses, including genetic adaptation and phenotypic plasticity [8], [9]. Most studies examining trait-environment relationships in plant communities have assigned a single, fixed trait value to each species, thereby accounting only for trait variation due to species turnover and ignoring intraspecific variation [10]. However, recent studies have shown that intraspecific variation may also play an important role in community trait responses to environmental variation. For example, Jung et al. [11] found that intraspecific variation accounted for up to 44% of the change in CWMs of several key functional traits across an elevation gradient in flood meadow communities. Similarly, Lepš et al. [10] found that community-level responses of multiple traits to fertilization and mowing in grassland communities were primarily driven by intraspecific responses. Results of these and other recent studies [12]–[14] demonstrate that accounting for intraspecific trait variation may be crucial for quantifying community trait responses to the environment, but the relative magnitude of intraspecific variation has varied strongly among and within studies. The next step beyond simply quantifying intraspecific trait variation is to understand the factors controlling its relative role and importance in communities. Determining when and where intraspecific variation matters at the community level is a major concern for plant ecology, with important implications for predicting community and ecosystem responses to global change [15].

One factor that may influence the relative importance of intraspecific trait variation is the spatial scale of the studied communities. Previous studies have shown that interspecific trait variation increases with increasing spatial scale due to species turnover driven by dispersal limitation and environmental filtering along gradients of increasing breadth [16], [17]. Intraspecific variation is also expected to increase with increasing spatial scale, as more genetic and plastic variability within species is included, but it is expected to saturate at large scales once the entire range and thus potential trait variation of individual species is reached [15], [18]. The relative magnitude of interspecific vs. intraspecific variation is therefore expected to increase at broad spatial scales encompassing strong environmental gradients [15]. This hypothesis has not been tested to our knowledge, in part due to the lack of studies measuring intraspecific variation at broad scales.

Another factor that may influence the relative importance of intraspecific trait variation is the type of environmental gradient considered. Previous studies have shown that intraspecific variation may be important for community responses to local-scale edaphic variation [8], [11], but its role in responses to broad-scale climatic variation has not been examined. Determining whether the relative importance of intraspecific variation differs between edaphic and climatic gradients is a useful step towards a more general understanding of when intraspecific variation matters at the community level. Knowledge of the role of intraspecific variation is also relevant for predicting responses of communities to climate change [19]. If community trait responses to climate are driven by species turnover, climate change will result in large changes in community composition and species distributions. On the other hand, if species are able to cope with climatic variation through genetic adaptation or phenotypic plasticity, community composition may remain stable [20], [21]. Assessing the degree to which intraspecific variation contributes to community trait responses to strong spatial climatic gradients will provide insights into which of these scenarios is most likely in the face of future climate change.

In this study, we examined community functional responses to environmental variation in old-field plant communities across eastern North America. The study was conducted on a broad spatial extent (1200 km in latitude), allowing us to test the relative importance of species turnover vs. intraspecific variation to community trait patterns along strong edaphic and climatic gradients at multiple spatial scales. Specifically, we addressed the following questions: 1) What is the relative importance of species turnover vs. intraspecific variation to among-site trait variation, and how is this influenced by spatial scale? We hypothesized that the relative importance of intraspecific trait variation would decrease with increasing spatial scale and breadth of environmental gradients. 2) How do community mean trait values respond to edaphic and climatic variation, and what are the relative contributions of species turnover and intraspecific variation to these responses? We hypothesized that intraspecific variation would be more important for community responses to edaphic compared to climatic factors.

## Materials and Methods

### Study site

We surveyed vegetation and functional traits in 22 old fields across the eastern United States in June-August, 2012. The study area extended from central South Carolina (30°40″N) to central New York (43°10″N), spanning approximately 1200 km of latitude (see Fig. S1 for map of study sites). The study sites were located on private and public land. A list of authorities who granted permission to conduct field work and should be contacted for future permissions is found in the Supporting Information (Table S5). The study area encompassed strong variation in both climatic and edaphic factors (Table 1), making it a useful system for comparing the influence of these factors on community functional composition. Moving from south to north, there was a strong decrease in mean annual temperature (17.9 to 6.9°C) and growing season length (263 to 156 annual frost-free days) and a weaker decrease in mean annual precipitation (1330 to 976 mm). In addition, with increasing latitude there was a strong increase in soil fertility and shift from coarse to fine-textured soils driven primarily by recent glaciation history [22].

**Table 1:** Mean, standard deviation, and range of environmental variables included in regression analyses.

Variable	Abbreviation	Unit	Mean	Standarddeviation	Range(max-min)
Mean annual temperature	MAT	°C	11.95	3.0	11.0
Mean annual precipitation	MAP	mm	1132	99.2	354
Cation exchange capacity	CEC	mEq kg^−1^	79.4	30.0	11.23
Soil pH	pH		5.6	0.37	1.5
Soil available phosphorus	P	mg kg^−1^	49.2	53.8	200.0
Soil available nitrogen	N	ppm	6.31	2.87	10.4
Soil organic matter	OM	%	4.98	1.73	7.38
Sand	Sa	%	41.75	18.82	71.91

The fields sampled had different histories of agricultural land use, but all had been abandoned for at least 5 years prior to sampling. Fields were maintained in early stages of succession by mowing in late summer or fall once every 1–2 years. Time since mowing was not significantly related to any environmental variable or response variable measured and was not included in the analyses. No burning, livestock grazing, or herbicide application had occurred in any of the fields within the past 5 years. Vegetation in the fields was almost entirely herbaceous, including a mix of grasses and forbs. Dominant species included goldenrods (e.g., *Solidago altissima*, *S. rugosa*) and grasses (e.g., *Andropogon virginicus*, *Schedonorus pratensis*, *Poa pratensis*). While there was considerable turnover in species composition across the study area, many of the dominant species were widely distributed, creating the potential for intraspecific variation to play an important role in community trait patterns.

### Vegetation and environmental data

In each field, we recorded the percent cover of vascular plant species in 20 1-m^2^ quadrats arrayed along transects. The number and arrangement of transects and spacing between quadrats varied depending on the size and shape of the field, with the goal of obtaining a representative sample of the overall vegetation. Cover values in the 20 quadrats were pooled to obtain the relative cover of each species in each field. We recorded a total of 227 species in the 22 sampled sites, with a mean richness of 21.4 species per site. Most species occurred in three or fewer fields, and there were large differences in species composition among sites (mean Bray-Curtis dissimilarity based on relative cover = 0.76; based on presence/absence = 0.75).

We collected soil samples in four randomly selected quadrats in each field and pooled samples for physical and chemical analysis. Percent sand, silt, and clay were determined using the hydrometer method [23]. Percent organic matter was measured as loss on ignition at 360°C. Soil samples were analyzed for cation exchange capacity (CEC), pH, available nitrogen (nitrate plus ammonium; KCl extraction/cadmium reduction method), available (Bray II) phosphorus [24], and available (Mehlich 3 extractant) sulfur, calcium, magnesium, potassium, iron, manganese, and aluminum [25]. Soil analyses were performed by Brookside Laboratories, Inc., New Bremen, OH, USA. We accessed daily precipitation and temperature data (1980–2010) for each site from Daymet (http://www.daymet.org). Using these data, we derived mean annual temperature, mean temperature of the coldest month, mean temperature of the warmest month, temperature seasonality (standard deviation of monthly mean temperature), annual frost-free days, annual growing-degree days (base of 5°C and cap of 30°C), annual precipitation, precipitation in the driest month, precipitation in the wettest month, and precipitation seasonality (CV of monthly precipitation). Environmental data were log transformed as necessary to improve normality.

### Trait data

We focused on four traits that relate to different aspects of plant functional strategy: vegetative height, leaf area, specific leaf area (SLA), and leaf dry matter content (LDMC). Vegetative height is related to light acquisition and competitive ability [26], [27]. Leaf area relates to energy and water balance and tolerance to environmental stress [28]. SLA is a central component of the leaf economics spectrum, which captures the tradeoff between rapid growth and resource conservation [29]. LDMC is also associated with the leaf economics spectrum as well as leaf water balance and resistance to physical stress [30], [31].

In each field, we measured functional traits of species that collectively accounted for 80–100% of the total vegetation cover. This sampling threshold has been shown to provide robust estimates of community mean trait values [32] but excludes some rare species. In each field, we selected five mature- and healthy-looking individuals of each species from different areas of the field for trait measurements. Vegetative height was measured as the distance (cm) from the base to the highest part of the general canopy of the plant. We selected one young, fully-expanded, upper canopy leaf per individual for leaf trait measurements. We measured the one-sided surface area and fresh mass of each leaf following full rehydration [33] and dry mass after oven drying at 80°C for 48 hours [31]. SLA was calculated as leaf area divided by dry mass (mm^2^/mg), and LDMC was calculated as leaf dry mass divided by fresh mass. We measured traits on 61 species total (mean 7.5 per field), representing on average 87% of the total cover of each field.

### Data analysis

Our first analysis partitioned the contributions of species turnover and intraspecific variation to among-site variation in functional traits following the approach of Lepš et al. [10]. For each field, we calculated three types of community-weighted mean trait values. 1) “Total CWM”, calculated as the abundance-weighted average of site-specific species mean trait values:
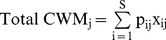
where p_ij_ is the relative cover of species i in site j, x_ij_ is the mean trait value of species i measured in site j, and S is the number of species sampled in the site. Among-site variation in total CWMs may be generated by a combination of changes in species composition (i.e. species turnover) and changes in species mean trait values (i.e. intraspecific variation) among sites. 2) “Interspecific CWM”, calculated as the abundance-weighted average of overall species mean trait values:



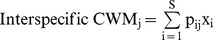
where x_i_ is the mean trait value of species i across all sites where it is measured. Variation in interspecific CWMs is generated by species turnover only. 3) “Intraspecific CWM”, calculated as the difference between the total CWM and interspecific CWM for a given site:



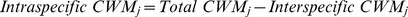



Variation in intraspecific CWMs is generated by intraspecific trait variation only. Using the total, interspecific, and intraspecific CWMs, we partitioned trait variation among sites into species turnover, intraspecific variation, and covariation components using the sum of squares decomposition approach of Lepš et al. [10]. Positive covariation indicates that species turnover and intraspecific variation reinforce each other (i.e., sites dominated by species with high trait values also have individuals with high trait values for their species), while negative covariation indicates that species turnover and intraspecific variation oppose each other (i.e., sites dominated by species with high trait values have individuals with low trait values for their species).

Our second analysis assessed community trait responses to edaphic and climatic variation and partitioned the contributions of species turnover and intraspecific variation to these responses. Due to strong correlations among variables, we attempted to reduce the dimensionality of the environmental data using principal components analysis, but the primary axes identified left much unexplained variation and were poorly related to community trait values. We therefore selected subsets of climatic (mean annual temperature and annual precipitation) and edaphic variables (sand, pH, CEC, organic matter, available nitrogen, and available phosphorus) that were expected to be important drivers of community functional structure and were not strongly correlated with each other (*r*<0.5). We modeled relationships between community-weighted mean trait values and edaphic and climatic factors using multiple linear regressions with the full subset of edaphic or climatic variables as predictors. We then performed stepwise model selection by AIC_c_ to select the best edaphic and climatic model for each trait. To quantify the contributions of species turnover, intraspecific variation, and their covariation to overall community trait responses, we partitioned the variance explained by the edaphic and climatic models using the sum of squares decomposition described above [10]. To examine community trait responses to specific environmental factors, we conducted a similar variance partitioning analysis using single environmental variables as predictors. We also used regression analyses to quantify and compare trait responses of the five most abundant and widely distributed species in the study area: forbs *Solidago altissima* and *Solidago rugosa*, and grasses *Schedonorus pratensis*, *Poa pratensis*, and *Andropogon virginicus*.

Our third analysis tested whether the between-site trait variation and the relative contribution of species turnover vs. intraspecific variation increased with increasing spatial and environmental distance. First, we calculated the geographic distance and environmental distance between each pair of sites in the study area (22 sites, resulting in 231 pairs). Geographic distance between sites was calculated as great circle distance and ranged from 6.5 to 1,151 km (see Fig. S2 for distribution of between-plot distances). Environmental distance was calculated as Euclidean distance using scaled environmental variables (Fig. S2). Next, for each pair of plots we calculated the total dissimilarity in CWMs, dissimilarity due to species turnover, and dissimilarity due to intraspecific variation by applying the sum of squares decomposition described above [10] to each plot pair. We quantified the relative importance of species turnover vs. intraspecific variation by taking the log of the ratio of the species turnover and intraspecific variation components. This created a symmetric measure of the relative contribution of species turnover vs. intraspecific variation to between-site trait dissimilarity, with positive values indicating a greater contribution of species turnover and negative values a greater contribution of intraspecific variation. We tested whether total between-site trait dissimilarity, dissimilarity due to species turnover, dissimilarity due to intraspecific variation, and the relative importance of turnover vs. intraspecific variation varied as a function of geographic and environmental distance using Mantel tests. Because geographic and environmental distance were strongly correlated (Mantel *r* = 0.70), we also used partial Mantel tests to test for the effect of either geographic or environmental distance while controlling for the other.

All analyses were conducted in R [34] using the fields [35], vegan [36], and ecodist [37] packages.

## Results

### Partitioning among-site trait variation

Among-site variation in community mean trait values was generated by a combination of species turnover and intraspecific variation, with species turnover making the greater contribution for each trait (Table 2). For height, SLA, and LDMC the contribution of species turnover was 2–2.5 times greater than that of intraspecific variation. For leaf area, almost all variation was due to species turnover (Table 2). There was positive covariation between species turnover and intraspecific variation for height and LDMC, indicating that the effects of species turnover and intraspecific variation reinforced each other (i.e., sites dominated by species with high values of those traits also tended to have individuals with high trait values for their species). There was little covariation between species turnover and intraspecific variation for leaf area or SLA (Table 2).

**Table 2:** Percentages of total variation in community-weighted mean trait values due to species turnover, intraspecific variation, and their covariation.

Trait	Speciesturnover	Intraspecificvariation	Covariation
Height	52%	22%	26%
Leaf area	89%	7%	4%
SLA	70%	28%	2%
LDMC	52%	27%	21%

Positive covariation indicates that sites dominated by species with high trait values also have individuals with higher than average trait values for their species.

### Community trait responses to soil and climate and relative contributions of species turnover vs. intraspecific variation

The best linear models relating environmental variables to community mean trait values varied among traits, but for all traits edaphic factors explained more variation (16–50%) than climatic factors (<20%; Table 3). For example, CWM height was best explained by a combination of soil pH, CEC, organic matter, and available phosphorus, and the best model for CWM leaf area included soil organic matter and available phosphorus (Table 3). CWM SLA was the trait most strongly influenced by climate, showing a significant decrease with increasing mean annual temperature (Table 3).

**Table 3:** Relationships between community-weighted mean trait values and environmental variables measured in the study.

Trait	Model	Predictors	*R* ^2^
Height	Edaphic	CEC (+), pH (−), OM (−), P (+)	0.50
	Climatic	MAP (–)	0.17
Leaf area	Edaphic	OM (−), P (−)	0.25
	Climatic	MAT (+)	0.16
SLA	Edaphic	pH (−), OM (+)	0.26
	Climatic	MAT (−)	0.20
LDMC	Edaphic	N (−), sand (+)	0.16
	Climatic	ns	-

Results are shown for the best edaphic and climatic models for each trait as determined by stepwise selection, including the predictor variables retained in each model and the direction of their effects (negative or positive) on the community trait value. Abbreviations: SLA, specific leaf area; LDMC, leaf dry matter content; predictor variables abbreviated as in Table 1.

**Figure 1:**
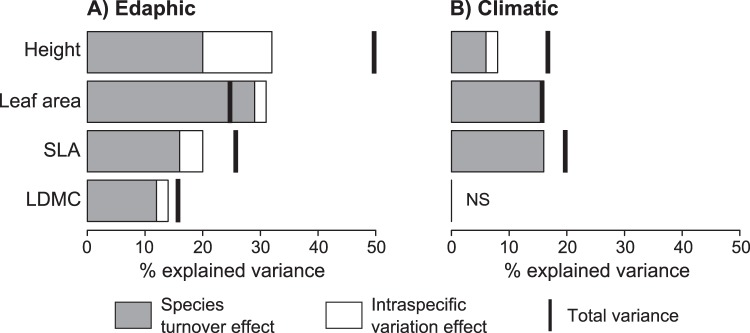
Decomposition of community trait responses to environment. Among-site variance in community-weighted mean trait values explained by (A) edaphic and (B) climatic variables is partitioned into species turnover, intraspecific variation, and covariation effects. Covariation is represented by the difference between the total variance and the sum of the species turnover and intraspecific variation effects. Total variance greater than the sum of species turnover and intraspecific variation effects indicates positive covariance. Total variance less than the sum of species turnover and intraspecific variation effects indicates negative covariance. Abbreviations: SLA, specific leaf area; LDMC, leaf dry matter content.

Community trait responses to the environment were generated primarily by species turnover (Fig. 1), but the importance of intraspecific variation depended on the trait and environmental factor. Intraspecific variation contributed most strongly to community responses of height. For all traits, intraspecific variation was more important, both in terms of total magnitude and relative to species turnover, for community responses to edaphic compared to climatic factors (Fig. 1). Similar results were obtained when using single environmental variables as predictors (Table S2). For example, the only cases in which intraspecific variation contributed more than species turnover were the responses of CWM height to soil CEC and available P (Table S2). Positive covariation between species turnover and intraspecific variation contributed strongly to community responses of height and to a lesser extent SLA, indicating that changes due to species turnover and intraspecific variation reinforced each other (Fig. 1). In contrast, there was weak or even negative covariation between species turnover and intraspecific variation effects for leaf area and LDMC.

**Figure 2:**
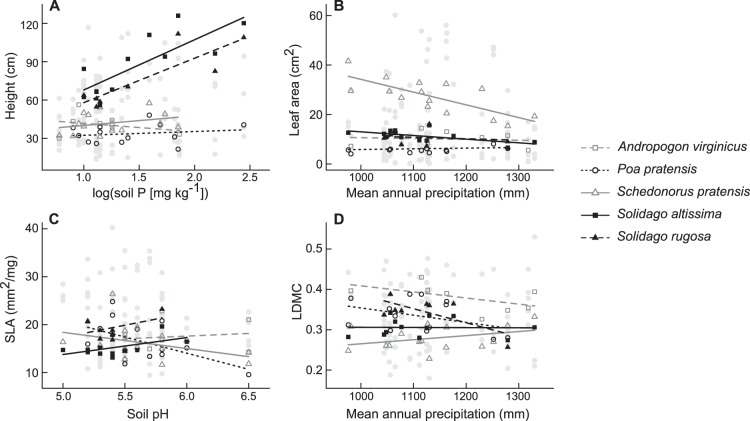
Intraspecific trait responses to environment. Examples of relationships between trait values of individual species and important environmental predictors are shown for each trait measured in the study: (A) height vs. soil P; (B) leaf area vs. mean annual precipitation; (C) specific leaf area vs. soil pH; (D) leaf dry matter content vs. mean annual precipitation. Points represent site-specific mean trait values of all species sampled in each site. Mean trait values and best fit lines from linear regressions are indicated for the five most abundant and widespread species in the study area: *Andropogon virginicus*, *Poa pratensis*, *Schedonorus pratensis*, *Solidago altissima*, and *Solidago rugosa*.

Individual species had strong trait responses to environmental variables in many cases, but these responses tended to be highly idiosyncratic, differing in strength and direction among species (Fig. 2; Table S3). There were no obvious patterns in the strength or consistency of intraspecific trait responses across species, traits, or environmental variables.

### Effects of geographic and environmental distance on between-site trait dissimilarity and species turnover vs. intraspecific variation effects

**Figure 3:**
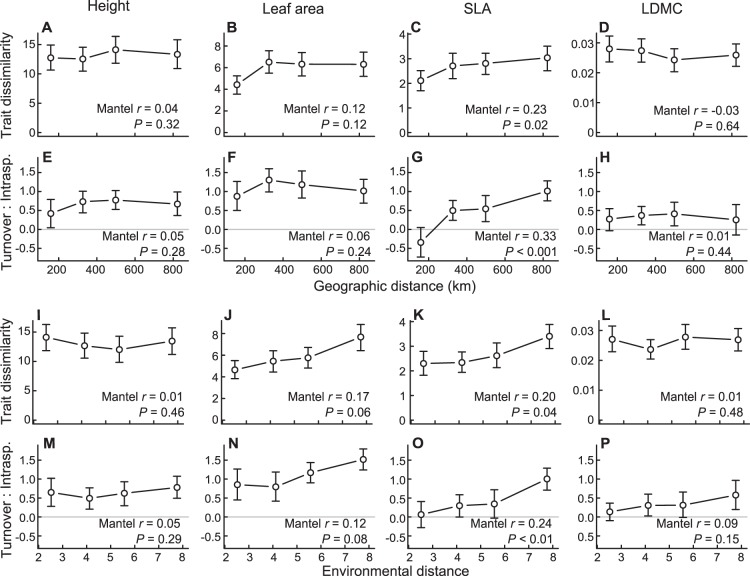
Effects of geographic and environmental distance on between-site variation in plant functional traits. Panels A-C and I-L show the trait dissimilarity (difference in community-weighted mean trait values) between sites as a function of geographic or environmental distance. Panels E-H and M-P show the relative contribution of species turnover vs. intraspecific variation (measured as log of species turnover effect divided by intraspecific variation effect) to the total trait dissimilarity between sites. Values were calculated for each pair of sites in the study area and binned by geographic or environmental distance (n = 57–58 pairs per bin) for ease of interpretation. Points represent mean values for each distance bin; error bars represent 95% bootstrap confidence intervals. Abbreviations: SLA, specific leaf area; LDMC, leaf dry matter content.

The influence of geographic and environmental distance on community trait dissimilarity varied among traits. Between-site dissimilarity in CWM SLA increased significantly with increasing geographic (*r* = 0.23; *P* = 0.02) and environmental distance (*r* = 0.20; *P* = 0.04; Fig. 3), indicating that nearby and environmentally similar sites had similar mean SLA. There was also a marginally significant increase in between-site dissimilarity in CWM leaf area with increasing environmental distance (*r* = 0.17; *P* = 0.07; Fig. 3). For both traits, increases in functional dissimilarity were driven by increases in species turnover effects, whereas intraspecific variation was insensitive to both geographic and environmental distance (Table S4). As a result, the relative importance of species turnover vs. intraspecific variation increased with increasing geographic (*r* = 0.33; *P*<0.01) and environmental distance (*r* = 0.24; *P*<0.01) for SLA and environmental distance for leaf area (*r* = 0.12; *P* = 0.08; Fig. 3). In contrast, between-site dissimilarity in CWMs and the relative importance of turnover vs. intraspecific variation were not related to geographic or environmental distance for height or LDMC (Fig. 3).

Due to the strong correlation between geographic and environmental distance, it was generally not possible to separate the effects of space vs. environment on community trait dissimilarity (partial Mantel tests not statistically significant; Table S4). The only exception was a positive relationship between geographic distance and the relative importance of species turnover for SLA even after removing the effect of environmental distance (partial *r* = 0.24; *P* = 0.01; Table S4).

## Discussion

Recent studies have shown that intraspecific trait variation may play a fundamental role in responses of plant communities to environmental variation [10]–[12], [19], but many questions remain about when intraspecific variation matters at the community level and the factors controlling its relative importance. By examining intraspecific trait variation in plant communities at an unprecedented spatial extent (1200 km), we were able to address unresolved questions about the relative role of intraspecific variation in community responses to the environment. We found that community-level trait variation was driven primarily by species turnover, but the relative importance of intraspecific variation depended strongly on the trait, environmental factor, and spatial scale considered. In particular, intraspecific variation was more important for responses to edaphic compared to climatic factors, and its relative importance decreased with increasing spatial scale and the related increase in breadth of environmental gradients for two of the four traits examined. These findings contribute to a more general understanding of the role intraspecific trait variation in plant communities, with implications for understanding community assembly and predicting community responses to global change.

### Role of intraspecific variation in community trait responses to environment

Community trait responses to environmental variation were primarily driven by species turnover, but intraspecific variation tended to play a larger role in community trait responses to edaphic compared to climatic variation. This finding may reflect differences in the spatial scale on which edaphic and climatic factors vary. Edaphic factors tend to vary on finer spatial scales than climatic factors [38], so the geographic ranges of individual species are likely to include a relatively larger proportion of the total edaphic variation than the total climatic variation found in a region. As a result of species being distributed widely along edaphic gradients but narrowly along climatic ones, there is greater potential for strong intraspecific trait responses to drive community-level trait shifts along edaphic gradients. In addition, fine-scale environmental heterogeneity is known to promote the evolution of adaptive phenotypic plasticity [39], [40], raising the possibility that plants have stronger plastic trait responses to fine-scale edaphic compared to broad-scale climatic factors. Assessing the strength of intraspecific trait responses to different environmental factors and how this relates to the spatial scale at which those factors vary is an important aim for future research.

The relative importance of intraspecific variation also varied among traits. The contribution of intraspecific variation to total among-site variation and responses to environmental factors was greatest for vegetative height. Intraspecific variation contributed to community-level variation in height directly and through strong positive covariation with species turnover effects, consistent with the expectation that interspecific and intraspecific trait responses should be in the same direction to increase individual plant fitness [8]. Intraspecific variation played a particularly large role in responses of height to edaphic factors such as available phosphorus. This finding is in line with previous studies showing that plant height is highly responsive to soil resource availability within species and that intraspecific variation contributes strongly to community-level variation in height [10], [41], [42]. In contrast, the relative contribution of intraspecific variation was smallest for leaf area and LDMC, traits that are known to be less plastic and for which intraspecific variation tends to be much smaller than interspecific variation [30], [43].

What explains the relatively weak contribution of intraspecific variation to most community-level trait-environment relationships observed in this study? The effect of intraspecific trait responses seen at the community level is the aggregate of responses of many individual species. Although traits of individual species responded strongly to the environment in many cases, these responses were highly idiosyncratic, differing in both strength and direction. As a result, they often cancelled out and generally failed to scale up to the community level. Previous studies examining trait variation in multiple species along an environmental gradient have also found intraspecific trait responses to be highly variable [12], [44], [45]. This variability may have several causes. First, species may respond to environmental variation by altering their multivariate functional strategies rather than single traits in isolation [19]. Functional tradeoffs can result in multiple trait combinations that are equally adaptive in a given environment [46], so responses of single traits may be variable. Second, traits respond not only to the abiotic environment, but also to biotic interactions [47]. The trait response of a particular species to an environmental change will therefore depend not only on the direct influence of the environment, but also on changes in biotic interactions mediated by traits of the target species and its neighbors [12]. Third, some traits may have unimodal responses to environmental gradients, such that trait values are maximized at a species’ environmental optimum. The observed direction of the intraspecific trait responses may therefore vary depending on the part of the gradient examined in relation to the environmental ranges and optima of the sampled species [44].

It should be noted that community trait-environment relationships observed in this study were generally weak, with much unexplained variation. This is probably due in part to a strong influence of site history on community assembly [48]. Sites with similar edaphic and climatic conditions were often dominated by functionally dissimilar species, suggesting that unknown past management regimes, along with dispersal history and stochastic effects may have played a large role in determining the functional composition of these early-successional communities. In addition, it is likely that key environmental variables, such as nitrogen supply rate and midsummer water potential, were not measured.

### Influence of spatial scale on the relative importance of species turnover vs. intraspecific variation

The broad spatial extent of our study allowed us to examine the influence of spatial scale on the relative importance of species turnover vs. intraspecific variation. Albert et al. [15] hypothesized that the relative importance of intraspecific variation should decrease with increasing spatial scale and the accompanying increase in environmental heterogeneity. We found limited support for this hypothesis for two of the four traits examined in the study, leaf area and SLA. Trait variation due to species turnover increased with increasing geographic and environmental distance for both traits, reflecting the effects of environmental filtering, dispersal limitation, or both [17]. In contrast, intraspecific trait variation was insensitive to both geographic and environmental distance, indicating that intraspecific variation in the studied communities was for the most part spatially random or driven by unmeasured fine-scale environmental variation. This agrees with the findings of Albert et al. [45] that most intraspecific variance in plant traits was found at fine spatial scales, i.e. within rather than between populations along an environmental gradient.

This study is the first to our knowledge to assess the contribution of intraspecific variation to community-level trait variation across a broad geographic extent encompassing strong climatic variation. The relative importance of intraspecific variation observed in this study was generally less than that observed for the same traits in previous local-scale studies of grassland communities. For example, Li et al. (unpublished data) showed that intraspecific variation was the primary source of community-level changes in height, SLA, and LDMC along a soil moisture gradient in subalpine meadow communities. Similarly, Kichenin et al. [12] found that intraspecific variation drove community shifts in SLA along an elevation gradient. At relatively small distances (<200 km), we also found that intraspecific variation contributed more than species turnover to among-site variation in SLA. For height, leaf area, and LDMC, species turnover was the primary source of between-community variation even at the smallest distances resolvable in this study (6.5 km), but it is possible that a similar transition from intraspecific variation to species turnover as the main source of among-community trait variation occurred at finer spatial scales.

### Implications

Our results have several potentially interesting implications for plant community ecology. First, with the growing recognition that intraspecific variation may play an important role in plant communities [49], and the great effort required to measure it [50], there is a need for information to help researchers decide when it should be considered in plant ecology studies. To this end, Albert et al. [15] proposed that intraspecific variation might be negligible and therefore safely ignored at very broad spatial scales encompassing strong environmental gradients. Our results provide limited support for this recommendation by showing for the first time that the relative importance of intraspecific variation in some traits decreases with increasing geographic and environmental distance between sites, so that species turnover dominates at very broad scales. In such cases, it may be acceptable to use species mean trait values to characterize community-level trait patterns, whereas measuring each species in each site where it occurs may be necessary for studies conducted on finer scales and shorter gradients [50]. Second, community trait-environment relationships are commonly used to infer community assembly mechanisms, particularly trait-mediated environmental filtering [8], but most studies have only considered interspecific trait variation. Our findings suggest that different environmental filters or selection pressures operate at different levels of organization within communities. Specifically, climatic factors may filter species based on their mean trait values, resulting in species turnover along broad-scale climatic gradients, whereas intraspecific variation may be more important for responses to edaphic filters. Third, the dominance of species turnover in driving community trait responses to spatial climatic gradients suggests that species turnover will also play a large role in responses of the studied communities to future predicted climate change [51].

To conclude, intraspecific trait variation may play an important role in community trait responses to the environment in some situations, and there is a need for empirical data to generalize when and to what extent it matters at the community level. We found that functional responses of old-field plant communities to environmental variation at broad spatial scales were primarily driven by species turnover, but several factors influenced the relative importance of intraspecific variation. Specifically, intraspecific variation was more important for responses of vegetative height compared to leaf traits, responses to edaphic compared to climatic gradients, and at fine compared to broad spatial scales. Future research should examine whether our findings extend to other plant communities and types of environmental gradients.

## Supporting Information

Figure S1 **Map of study sites and location of study area within North America.**
(DOCX)

Figure S2 **Distributions of geographic and environmental distances between study sites.**
(DOCX)

Table S1 Mean trait values of sampled species.
(DOCX)

Table S2 Decomposition of variation in community-weighted mean trait values explained by single environmental variables.
(DOCX)

Table S3 Slopes of intraspecific trait-environment relationships for the five most frequently occurring study species.
(DOCX)

Table S4 Results of Mantel and partial Mantel tests for effects of geographic and environmental distance on between-site trait dissimilarity.
(DOCX)

Table S5 List of authorities issuing permits for field work at study sites.
(DOCX)

## References

[1] Lavorel S, Garnier E (2002) Predicting changes in community composition and ecosystem functioning from plant traits: revisiting the Holy Grail. Funct Ecol 16: 545–556.

[2] McGill BJ, Enquist BJ, Weiher E, Westoby M (2006) Rebuilding community ecology from functional traits. Trends Ecol Evol 21: 178–185. 16701083 10.1016/j.tree.2006.02.002

[3] Suding KN, Lavorel S, Chapin FS III, Cornelissen JHC, et al (2008) Scaling environmental change through the community-level: a trait-based response-and-effect framework for plants. Glob Chang Biol 14: 1125–1140.

[4] Diaz S, Cabido M (2001) Vive la différence: plant functional diversity matters to ecosystem processes. Trends Ecol Evol 16: 646–655.

[5] Shipley B, Vile D, Garnier E (2006) From plant traits to plant communities: a statistical mechanistic approach to biodiversity. Science 314: 812–814. 17023613 10.1126/science.1131344

[6] Chapin FS III, Zavaleta ES, Eviner VT, Naylor RL, Vitousek PM, et al (2000) Consequences of changing biodiversity. Nature 405: 234–242. 10821284 10.1038/35012241

[7] Ricotta C, Moretti M (2011) CWM and Rao’s quadratic diversity: a unified framework for functional ecology. Oecologia 167: 181–188. 21424717 10.1007/s00442-011-1965-5

[8] Cornwell WK, Ackerly DD (2009) Community assembly and shifts in plant trait distributions across an environmental gradient in coastal California. Ecol Monogr 79: 109–126.

[9] Ackerly DD (2003) Community assembly, niche conservatism, and adaptive evolution in changing environments. Int J Plant Sci 164: S165–S184.

[10] Lepš J, de Bello F, Šmilauer P, Doležal J (2011) Community trait response to environment: disentangling species turnover vs intraspecific trait variability effects. Ecography 34: 856–863.

[11] Jung V, Violle C, Mondy C, Hoffmann L, Muller S (2010) Intraspecific variability and trait-based community assembly. J Ecol 98: 1134–1140.

[12] Kichenin E, Wardle DA, Peltzer DA, Morse CW, Freschet GT (2013) Contrasting effects of plant inter- and intraspecific variation on community-level trait measures along an environmental gradient. Funct Ecol 27: 1254–1261.

[13] Auger S, Shipley B (2012) Inter-specific and intra-specific trait variation along short environmental gradients in an old-growth temperate forest. J Veg Sci 24: 419–428.

[14] Pérez-Ramos I, Roumet C, Cruz P, Blanchard A, Autran P, et al (2012) Evidence for a “plant community economics spectrum” driven by nutrient and water limitations in a Mediterranean rangeland of southern France. J Ecol 100: 1315–1327.

[15] Albert CH, Grassein F, Schurr FM, Vieilledent G, Violle C (2011) When and how should intraspecific variability be considered in trait-based plant ecology? Perspect Plant Ecol Evol Syst 13: 217–225.

[16] Swenson NG, Anglada-Cordero P, Barone JA (2011) Deterministic tropical tree community turnover: evidence from patterns of functional beta diversity along an elevational gradient. Proc R Soc B 278: 877–884. 10.1098/rspb.2010.1369 PMC3049044 20861048

[17] Siefert A, Ravenscroft C, Weiser MD, Swenson NG (2013) Functional beta-diversity patterns reveal deterministic community assembly processes in eastern North American trees. Glob Ecol Biogeogr 22: 682–691.

[18] Violle C, Jiang L (2009) Towards a trait-based quantification of species niche. J Plant Ecol 2: 87–93.

[19] Jung V, Albert CH, Violle C, Kunstler G, Loucougaray G, et al (2014) Intraspecific trait variability mediates the response of subalpine grassland communities to extreme drought events. J Ecol 102: 45–53.

[20] Lloret F, Escudero A, Iriondo JM, Martínez-Vilalta J, Valladares F (2012) Extreme climatic events and vegetation: the role of stabilizing processes. Glob Chang Biol 18: 797–805.

[21] Grime JP, Fridley JD, Askew AP, Thompson K, Hodgson JG, et al (2008) Long-term resistance to simulated climate change in an infertile grassland. Proc Natl Acad Sci USA 105: 10028–10032. 18606995 10.1073/pnas.0711567105 PMC2481365

[22] Wright JP, Fridley JD (2010) Biogeographic synthesis of secondary succession rates in eastern North America. J Biogeogr 37: 1585–1596.

[23] Bouyoucos GJ (1962) Hydrometer method improved for making particle size analyses of soils. Agron J 54: 464–465.

[24] Bray RH, Kurtz LT (1945) Determination of total, organic, and available forms of phosphorus in soils. Soil Sci 59: 39–46.

[25] Mehlich A (1984) Mehlich 3 soil test extractant: A modification of Mehlich 2 extractant. Commun Soil Sci Plant Anal 15: 1409–1416.

[26] Gaudet CL, Keddy PA (1988) A comparative approach to predicting competitive ability from plant traits. Nature 334: 242–243.

[27] Wright IJ, Reich PB, Cornelissen JHC, Falster DS, Groom PK, et al (2005) Modulation of leaf economic traits and trait relationships by climate. Glob Ecol Biogeogr 14: 411–421.

[28] Ackerly D, Reich P (1999) Convergence and correlations among leaf size and function in seed plants: a comparative test using independent contrasts. Am J Bot 86: 1272–1281. 10487815

[29] Wright IJ, Reich PB, Westoby M, Ackerly DD, Baruch Z, et al (2004) The worldwide leaf economics spectrum. Nature 428: 821–827. 15103368 10.1038/nature02403

[30] Wilson P, Thompson K, Hodgson J (1999) Specific leaf area and leaf dry matter content as alternative predictors of plant strategies. New Phytol 143: 155–162.

[31] Perez-Harguindeguy N, Diaz S, Garnier E, Lavorel S, Poorter H, et al (2013) New handbook for standardised measurement of plant functional traits worldwide. Aust J Bot 61: 167–234.

[32] Pakeman RJ, Lepš J, Kleyer M, Lavorel S, Garnier E (2009) Relative climatic, edaphic and management controls of plant functional trait signatures. J Veg Sci 20: 148–159.

[33] Garnier E, Shipley B, Roumet C, Laurent G (2001) A standardized protocol for the determination of specific leaf area and leaf dry matter content. Funct Ecol 15: 688–695.

[34] R Core Development Team (2012) R: A Language and Environment for Statistical Computing. Available: http://www.r-project.org.

[35] Furrer R, Nychka D, Sain S (2012) fields: Tools for spatial data. Available: http://cran.r-project.org/package=fields.

[36] Oksanen J, Blanchet FG, Kindt R, Legendre P, Minchin P, et al. (2012) vegan: Community ecology package. Available: http://cran.r-project.org/package=vegan.

[37] Goslee S, Urban D (2007) The ecodist package for dissimilarity-based analysis of ecological data. J Stat Softw 22: 1–19.

[38] Lechowicz MJ, Bell G (1991) The ecology and genetics of fitness in forest plants II. Microspatial heterogeneity of the edaphic environment. J Ecol 79: 687–696.

[39] Via S, Lande R (1985) Genotype-environment interaction and the evolution of phenotypic plasticity. Evolution 39: 505–522. 28561964 10.1111/j.1558-5646.1985.tb00391.x

[40] Baythavong BS (2011) Linking the spatial scale of environmental variation and the evolution of phenotypic plasticity: selection favors adaptive plasticity in fine-grained environments. Am Nat 178: 75–87. 21670579 10.1086/660281

[41] Dantas VDL, Pausas JG, Batalha MA, Loiola PDP, Cianciaruso MV (2013) The role of fire in structuring trait variability in Neotropical savannas. Oecologia 171: 487–494. 22926723 10.1007/s00442-012-2431-8

[42] Gross N, Börger L (2013) Uncovering multiscale effects of aridity and biotic interactions on the functional structure of Mediterranean shrublands. J Ecol: 637–649.

[43] Roche P, Díaz-Burlinson N, Gachet S (2004) Congruency analysis of species ranking based on leaf traits: which traits are the more reliable? Plant Ecol 174: 37–48.

[44] Albert CH, Thuiller W, Yoccoz NG, Soudant A, Boucher F, et al (2010) Intraspecific functional variability: extent, structure and sources of variation. J Ecol 98: 604–613.

[45] De Frenne P, Graae B (2013) Latitudinal gradients as natural laboratories to infer species’ responses to temperature. J Ecol 101: 784–795.

[46] Marks CO, Lechowicz MJ (2006) Alternative designs and the evolution of functional diversity. Am Nat 167: 55–66. 16475099 10.1086/498276

[47] Callaway RRM, Pennings SSC, Richards CLC (2003) Phenotypic plasticity and interactions among plants. Ecology 84: 1115–1128.

[48] Chase JM (2003) Community assembly: when should history matter? Oecologia 136: 489–498. 12836009 10.1007/s00442-003-1311-7

[49] Violle C, Enquist BJ, McGill BJ, Jiang L, Albert CH, et al (2012) The return of the variance: intraspecific variability in community ecology. Trends Ecol Evol 27: 244–252. 22244797 10.1016/j.tree.2011.11.014

[50] Baraloto C, Paine CET, Patiño S, Bonal D, Hérault B, et al (2010) Functional trait variation and sampling strategies in species-rich plant communities. Funct Ecol 24: 208–216.

[51] Northeast Climate Impacts Assessment (2006) Climate change in the US Northeast. Union of Concerned Scientists Publications, Cambridge, MA.

