# Supplementary figures and images for: Community Functional Responses to Soil and Climate at Multiple Spatial Scales: When Does Intraspecific Variation Matter?
Source: PLoS One. 2014 Oct 20;9(10):e111189. doi: 10.1371/journal.pone.0111189 (PMC4203824; doi:10.1371/journal.pone.0111189)

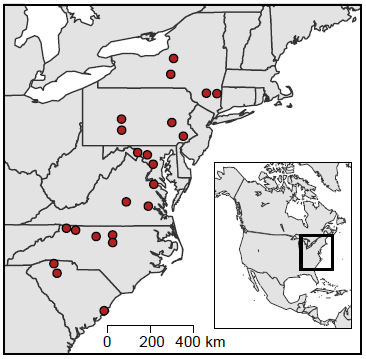


**Figure S1. Map of study sites and location of study area within North America.**

Supplement: Figure S1 — Map of study sites and location of study area within North America. (DOCX) [file pone.0111189.s001.docx]
