# Supplementary material for: Community Functional Responses to Soil and Climate at Multiple Spatial Scales: When Does Intraspecific Variation Matter?
Source: PLoS One. 2014 Oct 20;9(10):e111189. doi: 10.1371/journal.pone.0111189 (PMC4203824; doi:10.1371/journal.pone.0111189)
Supplement: Figure S2 — Distributions of geographic and environmental distances between study sites. (DOCX) [file pone.0111189.s002.docx]

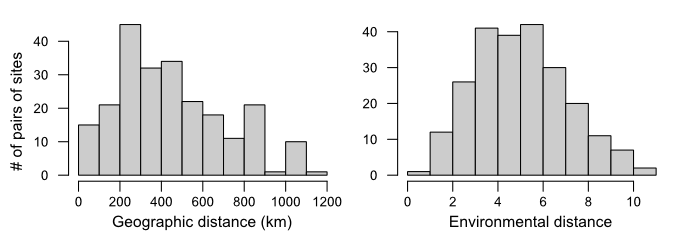


**Figure S2. Distribution of geographic and environmental distances between pairs of sites in the study area.**
