# Supplementary material for: Community Functional Responses to Soil and Climate at Multiple Spatial Scales: When Does Intraspecific Variation Matter?
Source: PLoS One. 2014 Oct 20;9(10):e111189. doi: 10.1371/journal.pone.0111189 (PMC4203824; doi:10.1371/journal.pone.0111189)
Supplement: Table S1 — Mean trait values of sampled species. (DOCX) [file pone.0111189.s003.docx]

**Table S1. Mean trait values of sampled species.**

| Species | Height (cm) | Leaf area (cm^2^) | SLA (mm^2^ mg^-1^) | LDMC |  | Species | Height (cm) | Leaf area (cm^2^) | SLA (mm^2^ mg^-1^) | LDMC |
| --- | --- | --- | --- | --- | --- | --- | --- | --- | --- | --- |
| *Achillea millefolium* | 19.8 | 15.2 | 11.9 | 0.28 |  | *Panicum anceps* | 56.6 | 46.6 | 20.0 | 0.32 |
| *Ambrosia artemisiifolia* | 52.1 | 16.7 | 24.1 | 0.24 |  | *Panicum virgatum* | 91.2 | 24.6 | 12.6 | 0.41 |
| *Andropogon virginicus* | 42.0 | 11.6 | 20.6 | 0.36 |  | *Paspalum notatum* | 37.9 | 7.0 | 16.6 | 0.33 |
| *Anthoxanthum odoratum* | 23.6 | 9.8 | 31.7 | 0.29 |  | *Phleum pratense* | 71.6 | 11.9 | 24.5 | 0.31 |
| *Apocynum cannabinum* | 112.0 | 13.1 | 13.5 | 0.37 |  | *Pityopsis graminifolia* | 20.4 | 9.9 | 13.4 | 0.31 |
| *Bromus inermis* | 61.3 | 9.6 | 16.9 | 0.39 |  | *Plantago lanceolata* | 18.2 | 14.2 | 16.9 | 0.21 |
| *Campsis radicans* | 51.8 | 35.5 | 15.3 | 0.35 |  | *Poa pratensis* | 33.6 | 6.2 | 16.4 | 0.33 |
| *Carex hirsutella* | 36.0 | 5.1 | 16.8 | 0.44 |  | *Prunella vulgaris* | 21.7 | 1.9 | 23.2 | 0.33 |
| *Carex* sp. | 22.3 | 7.6 | 21.1 | 0.24 |  | *Rubus cuneifolius* | 51.2 | 22.4 | 11.6 | 0.42 |
| *Carex swanii* | 27.2 | 6.1 | 25.9 | 0.45 |  | *Rubus* sp. | 32.9 | 33.1 | 13.9 | 0.40 |
| *Centaurea jacea* | 70.6 | 3.1 | 14.6 | 0.32 |  | *Schedonorus pratensis* | 42.0 | 27.2 | 16.1 | 0.28 |
| *Centaurea nigra* | 51.6 | 4.1 | 20.8 | 0.24 |  | *Schizacyrium scoparium* | 65.2 | 24.0 | 15.5 | 0.34 |
| *Clinopodium vulgare* | 26.8 | 3.2 | 26.5 | 0.25 |  | *Scleria pauciflora* | 32.5 | 5.1 | 20.2 | 0.37 |
| *Coronilla varia* | 51.6 | 6.4 | 23.5 | 0.23 |  | *Setaria pumila* | 69.6 | 15.6 | 25.7 | 0.24 |
| *Dactylis glomerata* | 40.5 | 27.6 | 25.2 | 0.28 |  | *Setaria* sp. | 44.3 | 4.8 | 25.4 | 0.30 |
| *Danthonia spicata* | 22.0 | 4.3 | 17.7 | 0.45 |  | *Solidago altissima* | 80.2 | 11.0 | 15.2 | 0.30 |
| *Daucus carota* | 68.2 | 15.0 | 12.1 | 0.22 |  | *Solidago gigantea* | 104.3 | 12.4 | 17.0 | 0.37 |
| *Desmodium obtusum* | 46.6 | 5.3 | 16.8 | 0.34 |  | *Solidago juncea* | 75.2 | 9.6 | 13.6 | 0.35 |
| *Dichanthelium aciculare* | 17.4 | 1.0 | 17.8 | 0.45 |  | *Solidago rugosa* | 76.5 | 10.7 | 19.2 | 0.35 |
| *Dichanthelium acuminatum* | 27.9 | 3.7 | 22.9 | 0.38 |  | *Sorghum halepense* | 116.8 | 56.1 | 19.9 | 0.32 |
| *Dichanthelium clandestinum* | 58.2 | 20.5 | 26.5 | 0.29 |  | *Symphiotrichum* sp. 1 | 61.3 | 1.1 | 13.8 | 0.31 |
| *Dichanthelium scoparium* | 72.2 | 7.9 | 23.5 | 0.33 |  | *Symphiotrichum* sp. 2 | 82.2 | 12.6 | 19.1 | 0.30 |
| *Doellingeria umbellata* | 76.0 | 11.7 | 13.9 | 0.38 |  | *Tephrosia florida* | 24.8 | 4.2 | 15.6 | 0.44 |
| *Elymus virginicus* | 88.8 | 15.1 | 19.3 | 0.44 |  | *Tephrosia spicata* | 16.8 | 17.1 | 11.8 | 0.38 |
| *Euthamia graminifolia* | 66.2 | 5.0 | 15.2 | 0.38 |  | *Trifolium repens* | 16.8 | 7.2 | 22.3 | 0.26 |
| *Festuca rubra* | 38.2 | 3.8 | 13.3 | 0.34 |  | Unk grass 1 | 20.7 | 1.9 | 17.5 | 0.48 |
| *Fragaria virginiana* | 17.1 | 23.3 | 14.1 | 0.37 |  | Unk grass 2 | 24.8 | 1.8 | 35.4 | 0.37 |
| *Galium mollugo* | 46.7 | 0.3 | 39.5 | 0.21 |  | Unk grass 3 | 27.8 | 7.8 | 32.9 | 0.28 |
| *Helianthemum rosmarinifolium* | 28.0 | 1.3 | 9.4 | 0.39 |  | Unk legume | 28.6 | 1.5 | 25.0 | 0.43 |
| *Holcus lanatus* | 51.1 | 7.8 | 30.8 | 0.25 |  | *Verbesina alternifolia* | 100.9 | 38.0 | 20.0 | 0.22 |
| *Lespedeza cuneata* | 60.8 | 2.1 | 18.9 | 0.47 |  | *Verbesina occidentalis* | 68.8 | 41.2 | 19.1 | 0.27 |
| *Lonicera* sp. | 39.9 | 7.9 | 23.8 | 0.29 |  |  |  |  |  |  |
