# Supplementary material for: Community Functional Responses to Soil and Climate at Multiple Spatial Scales: When Does Intraspecific Variation Matter?
Source: PLoS One. 2014 Oct 20;9(10):e111189. doi: 10.1371/journal.pone.0111189 (PMC4203824; doi:10.1371/journal.pone.0111189)
Supplement: Table S2 — Decomposition of variation in community-weighted mean trait values explained by single environmental variables. (DOCX) [file pone.0111189.s004.docx]

**Table S2. Decomposition of variation in community-weighted mean trait values explained by single environmental variables.** Total among-site variance is decomposed into species turnover, intraspecific variation, and covariation components. Values shown are percentages of total among-site variance.

| Predictor | Vegetative height | | | Leaf area | | | SLA | | | LDMC | | |
| --- | --- | --- | --- | --- | --- | --- | --- | --- | --- | --- | --- | --- |
|  | Turn. | Intra. | Cov. | Turn. | Intra. | Cov. | Turn. | Intra. | Cov. | Turn. | Intra. | Cov. |
| Mean annual temp. (°C) | 4.5% | 0.2% | 1.8% | 16.1% | 0.0% | -0.4% | 16.1% | 0.3% | 4.0% | 3.8% | 0.6% | -3.1% |
| Mean annual precip. (mm) | 7.3% | 2.6% | 8.8% | 1.3% | 1.0% | -2.2% | 1.1% | 0.3% | -1.1% | 1.2% | 0.0% | -0.2% |
| Soil CEC (mEq kg^-1^) | 0.5% | 4.2% | 2.9% | 4.7% | 0.6% | -3.3% | 0.2% | 0.0% | 2.7% | 4.3% | 0.1% | 1.7% |
| Soil pH | 4.8% | 0.3% | -2.5% | 2.9% | 0.0% | 0.6% | 4.3% | 4.5% | 8.7% | 0.0% | 0.0% | 0.0% |
| Soil organic matter (%) | 0.0% | 0.1% | 0.0% | 9.3% | 0.1% | 1.8% | 14.8% | 0.1% | 1.8% | 7.9% | 0.1% | -2.1% |
| Soil available P (mg kg^-1^) | 4.8% | 9.5% | 13.5% | 11.1% | 1.3% | -7.6% | 2.4% | 0.6% | 2.5% | 0.6% | 0.5% | -1.2% |
| Soil available N (ppm) | 2.5% | 1.1% | -3.3% | 0.0% | 0.1% | -0.1% | 0.0% | 0.7% | 0.1% | 3.1% | 1.1% | 3.6% |
| Soil sand content (%) | 2.4% | 1.0% | 3.1% | 2.1% | 0.7% | -2.4% | 4.2% | 0.9% | -3.9% | 5.3% | 0.2% | -2.0% |
