# Supplementary material for: Community Functional Responses to Soil and Climate at Multiple Spatial Scales: When Does Intraspecific Variation Matter?
Source: PLoS One. 2014 Oct 20;9(10):e111189. doi: 10.1371/journal.pone.0111189 (PMC4203824; doi:10.1371/journal.pone.0111189)
Supplement: Table S3 — Slopes of intraspecific trait-environment relationships for the five most frequently occurring study species. (DOCX) [file pone.0111189.s005.docx]

**Table S3. Slopes of intraspecific trait-environment relationships for the five most frequently occurring species in the study area.** *Andropogon virginicus* (n = 8 sites), *Poa pratensis* (n = 13), *Schedonorus pratensis* (n = 15), *Solidago altissima* (n = 12), and *Solidago rugos*a (n = 8). Environmental variables were scaled to mean = 0 and sd = 1 to allow comparison of slopes among variables.

| Trait and species | Mean annual temp. (°C) | Mean annual precip. (mm) | CEC (mEq kg^-1^) | pH | Organic matter (%) | Available P (mg kg^-1^) | Available N (ppm) | Sand (%) |
| --- | --- | --- | --- | --- | --- | --- | --- | --- |
| Vegetative height |  |  |  |  |  |  |  |  |
| *A. virginicus* | -4.84 | -1.52 | -0.92 | -0.91 | 1.00 | -2.95 | -1.91 | -3.73 |
| *P. pratensis* | 2.79 | -4.35 | 2.69 | 3.27 | -0.70 | 1.32 | 1.19 | -3.84 |
| *S. pratensis* | -0.90 | -5.78 | 3.73 | 2.48 | -4.85 | 3.36 | -0.47 | -2.23 |
| *S. altissima* | -4.17 | -10.10 | 8.88 | 2.06 | -3.66 | 17.47 | 3.58 | -13.23 |
| *S. rugosa* | -3.57 | -4.40 | 0.69 | -10.38 | -1.94 | 15.44 | 5.72 | 0.35 |
| Leaf area |  |  |  |  |  |  |  |  |
| *A. virginicus* | -1.82 | -0.34 | -0.05 | -0.45 | 0.92 | -2.50 | -1.04 | -1.38 |
| *P. pratensis* | -0.99 | 0.27 | -0.69 | -1.26 | 0.32 | 1.25 | 0.97 | 0.90 |
| *S. pratensis* | 1.62 | -5.11 | 3.59 | 0.95 | -3.42 | 2.90 | -1.85 | -3.17 |
| *S. altissima* | -1.04 | -1.47 | 0.86 | -0.50 | -0.21 | 1.27 | 0.19 | -1.05 |
| *S. rugosa* | -1.06 | -0.57 | 0.94 | -0.46 | 0.37 | 1.39 | 0.62 | -1.57 |
| Specific leaf area |  |  |  |  |  |  |  |  |
| *A. virginicus* | -1.22 | 0.55 | 0.77 | 0.41 | 1.44 | -0.98 | 1.36 | -0.06 |
| *P. pratensis* | -2.86 | 2.15 | -0.21 | -2.52 | 2.23 | 0.78 | 0.37 | 1.22 |
| *S. pratensis* | -1.70 | -0.98 | 0.32 | -1.26 | -0.17 | 3.21 | -0.67 | -0.38 |
| *S. altissima* | -0.11 | 0.58 | 0.01 | 1.32 | 0.33 | -0.17 | 0.23 | -0.56 |
| *S. rugosa* | 0.32 | 1.12 | -0.25 | 1.90 | 0.68 | -0.13 | 0.99 | 0.76 |
| Leaf dry matter content |  |  |  |  |  |  |  |  |
| *A. virginicus* | -0.015 | -0.015 | -0.002 | -0.014 | -0.006 | -0.024 | -0.030 | -0.016 |
| *P. pratensis* | 0.021 | -0.018 | 0.001 | 0.015 | -0.014 | -0.005 | -0.012 | -0.014 |
| *S. pratensis* | 0.013 | 0.010 | -0.007 | 0.001 | 0.007 | -0.025 | -0.003 | 0.003 |
| *S. altissima* | -0.009 | 0.000 | 0.006 | -0.008 | 0.000 | 0.011 | 0.001 | 0.007 |
| *S. rugosa* | -0.015 | -0.035 | 0.011 | -0.053 | -0.008 | 0.009 | -0.011 | -0.014 |
