# Supplementary material for: Community Functional Responses to Soil and Climate at Multiple Spatial Scales: When Does Intraspecific Variation Matter?
Source: PLoS One. 2014 Oct 20;9(10):e111189. doi: 10.1371/journal.pone.0111189 (PMC4203824; doi:10.1371/journal.pone.0111189)
Supplement: Table S4 — Results of Mantel and partial Mantel tests for effects of geographic and environmental distance on between-site trait dissimilarity. (DOCX) [file pone.0111189.s006.docx]

**Table S4. Results of Mantel and partial Mantel tests for relationships between geographic and environmental distance and trait dissimilarity between study sites.**

|  | Geographic | | Environmental | | Geo \| Env | | Env \| Geo | |
| --- | --- | --- | --- | --- | --- | --- | --- | --- |
| Response | Mantel *r* | *P* | Mantel *r* | *P* | Mantel *r* | *P* | Mantel *r* | *P* |
| Height |  |  |  |  |  |  |  |  |
| Total dissimilarity | 0.04 | 0.31 | 0.00 | 0.47 | 0.05 | 0.33 | -0.03 | 0.64 |
| Species turnover | -0.02 | 0.54 | 0.02 | 0.38 | -0.04 | 0.67 | 0.04 | 0.29 |
| Intraspecific variation | 0.02 | 0.39 | 0.04 | 0.34 | 0.00 | 0.54 | 0.03 | 0.40 |
| Turnover:Intraspecific | 0.05 | 0.28 | 0.05 | 0.29 | 0.02 | 0.38 | 0.02 | 0.39 |
| Leaf area |  |  |  |  |  |  |  |  |
| Total dissimilarity | 0.12 | 0.11 | 0.17 | 0.07 | 0.00 | 0.51 | 0.12 | 0.18 |
| Species turnover | 0.16 | 0.05 | 0.22 | 0.03 | 0.01 | 0.48 | 0.15 | 0.09 |
| Intraspecific variation | 0.09 | 0.15 | 0.05 | 0.28 | 0.08 | 0.22 | -0.02 | 0.59 |
| Turnover:Intraspecific | 0.06 | 0.24 | 0.12 | 0.08 | -0.04 | 0.67 | 0.12 | 0.12 |
| SLA |  |  |  |  |  |  |  |  |
| Total dissimilarity | 0.23 | 0.02 | 0.20 | 0.04 | 0.13 | 0.14 | 0.05 | 0.31 |
| Species turnover | 0.39 | <0.01 | 0.21 | 0.04 | 0.35 | 0.00 | -0.10 | 0.78 |
| Intraspecific variation | -0.08 | 0.76 | -0.04 | 0.61 | -0.08 | 0.75 | 0.03 | 0.40 |
| Turnover:Intraspecific | 0.33 | <0.01 | 0.24 | <0.01 | 0.24 | 0.01 | 0.01 | 0.52 |
| LDMC |  |  |  |  |  |  |  |  |
| Total dissimilarity | -0.03 | 0.63 | 0.00 | 0.48 | -0.05 | 0.69 | 0.03 | 0.34 |
| Species turnover | 0.03 | 0.36 | 0.12 | 0.13 | -0.08 | 0.74 | 0.14 | 0.10 |
| Intraspecific variation | 0.01 | 0.42 | -0.07 | 0.77 | 0.09 | 0.19 | -0.11 | 0.87 |
| Turnover:Intraspecific | 0.01 | 0.43 | 0.09 | 0.15 | -0.07 | 0.78 | 0.12 | 0.11 |
