# Supplementary material for: Community Functional Responses to Soil and Climate at Multiple Spatial Scales: When Does Intraspecific Variation Matter?
Source: PLoS One. 2014 Oct 20;9(10):e111189. doi: 10.1371/journal.pone.0111189 (PMC4203824; doi:10.1371/journal.pone.0111189)
Supplement: Table S5 — List of authorities issuing permits for field work at study sites. (DOCX) [file pone.0111189.s007.docx]

**Table S5. List of authorities issuing permits for field work at study sites.**

| **Site name** | **Location** | **Authority issuing permit** | **Contact** |
| --- | --- | --- | --- |
| Hampton Plantation SHS | McClellanville, SC | South Carolina Department of Natural Resources | 1000 Assembly Street, Columbia, SC 29201 |
| Musgrove Mill SHS | Clinton, SC | South Carolina Department of Natural Resources | 1000 Assembly Street, Columbia, SC 29201 |
| VaDuMar Park | Boiling Springs, SC | Spartanburg County Parks Department | 9039 Fairforest Rd. Spartanburg, SC 29301 |
| New River SP | Laurel Springs, NC | North Carolina Division of Parks and Recreation | 1615 MSC, Raleigh, NC 27699 |
| Stone Mountain SP | Roaring Gap, NC | North Carolina Division of Parks and Recreation | 1615 MSC, Raleigh, NC 27699 |
| Eno River SP | Durham, NC | North Carolina Division of Parks and Recreation | 1615 MSC, Raleigh, NC 27699 |
| Guilford Courthouse NMP | Greensboro, NC | U.S. National Park Service | 1849 C Street NW, Washington, DC 20240 |
| Duke Forest | Durham, NC | Duke University | Box 90328, Duke University, Durham, NC 27708 |
| Cumberland SF | Cumberland, VA | Virginia Department of Forestry | 900 Natural Resources Drive, Charlottesville, VA 22903 |
| Ingrid & Walter Rice Center | Charles City, VA | Virginia Commonwealth University | 1000 West Cary St., P.O Box 842012, Richmond, VA 23284 |
| George Washington Birthplace NM | Colonial Beach, VA | U.S. National Park Service | 1849 C Street NW, Washington, DC 20240 |
| Sky Meadows SP | Delaplane, VA | Virginia Department of Conservation and Recreation | 600 E. Main St., 24^th^ Floor, Richmond, VA 23219 |
| Monocacy NB | Frederick, MD | U.S. National Park Service | 1849 C Street NW, Washington, DC 20240 |
| Antietam NB | Sharpsburg, MD | U.S. National Park Service | 1849 C Street NW, Washington, DC 20240 |
| Hoxie Gorge Nature Preserve | Blodgett Mills, NY | State University of New York at Cortland | 38 Graham Ave, Cortland, NY 13045 |
| Cornell Biological Field Station | Bridgeport, NY | Cornell University | 900 Shackelton Point Rd., Bridgeport, NY 13030 |
| Allegheny Portage Railroad NHS | Gallitzin, PA | U.S. National Park Service | 1849 C Street NW, Washington, DC 20240 |
| Prince Gallitzin SP | Patton, PA | Pennsylvania Department of Conservation and Natural Resources | 400 Market St., Harrisburg, PA 17101 |
| Valley Forge NHP | King of Prussia, PA | U.S. National Park Service | 1849 C Street NW, Washington, DC 20240 |
| Hawk Mountain Sanctuary | Kempton, PA | Hawk Mountain Sanctuary | 1700 Hawk Mountain Rd, Kempton, PA 19529 |
| Cary Institute | Millbrook, NY | Cary Institute of Ecosystem Studies | 2801 Sharon Turnpike, Millbrook, New York 12545 |
| Mohonk Preserve | Gardiner, NY | Mohonk Preserve | 3197 Rte 44 55, Gardiner, NY 12525 |
